# Supplementary material for: Strain control of a bandwidth-driven spin reorientation in Ca3Ru2O7
Source: Nat Commun. 2023 Oct 4;14:6197. doi: 10.1038/s41467-023-41714-8 (PMC10550943; doi:10.1038/s41467-023-41714-8)
Supplement: Supplementary file 1 — Supplementary Information [file 41467_2023_41714_MOESM1_ESM.pdf]

Supplementary Information for "Strain control of a bandwidth-driven spin reorientation in $Ca_3Ru_2O_7$"

C. D. Dashwood,[1] A. H. Walker,[1] M. P. Kwasigroch,[2,3] L. S. I. Veiga,[1,4] Q. Faure,[1,5] J. G. Vale,[1] D. G. Porter,[4] P. Manuel,[6] D. D. Khalyavin,[6] F. Orlandi,[6] C. V. Colin,[7] O. Fabelo,[8] F. Krüger,[1,6] R. S. Perry,[1] R. D. Johnson,[9] A. G. Green,[1] and D. F. McMorrow[1]

[1]*London Centre for Nanotechnology and Department of Physics and Astronomy, University College London, London, WC1E 6BT, United Kingdom*

[2]*Department of Mathematics, University College London, London, WC1H 0AY, United Kingdom*

[3]*Trinity College, Cambridge, CB2 1TQ, United Kingdom*

[4]*Diamond Light Source, Harwell Science and Innovation Campus, Didcot, Oxfordshire, OX11 0DE, United Kingdom*

[5]*Laboratoire León Brillouin, CEA, CNRS, Université Paris-Saclay, CEA-Saclay, 91191 Gif-sur-Yvette, France*

[6]*ISIS Neutron and Muon Source, STFC Rutherford Appleton Laboratory, Didcot, Oxfordshire, OX11 0QX, United Kingdom*

[7]*Université Grenoble Alpes, CNRS, Institut Néel, 38000 Grenoble, France*

[8]*Institut Laue-Langevin, 71 Avenue des Martyrs, CS 20156, 38042 Grenoble, France*

[9]*Department of Physics and Astronomy, University College London, London, WC1E 6BT, United Kingdom*

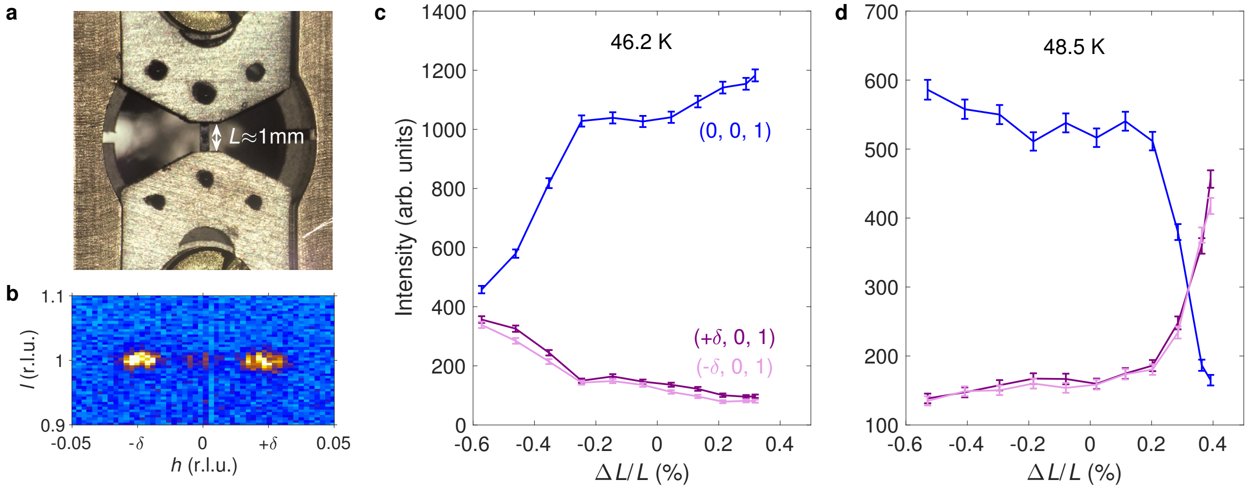

Supplementary Fig. 1. **Neutron scattering under stress.** **a** Image of the bar-shaped $Ca_3Ru_2O_7$ sample mounted over a distance $L \approx 1\,\text{mm}$ between the sample plates of the CS200T cell. **b** $(h, 0, l)$ reciprocal space map at 47.7 K and zero strain showing satellite peaks at $(\pm\delta, 0, 1)$ around the commensurate $(0, 0, 1)$ position. **c–d** Integrated intensity of the commensurate $(0, 0, 1)$ and incommensurate $(\delta, 0, 1)$ peaks as a function of applied strain at 46.2 K and 48.5 K. Errors are standard deviations.

SUPPLEMENTARY NOTE 1: ADDITIONAL NEUTRON SCATTERING DATA

In the main text, we present neutron scattering data taken on a strained $Ca_3Ru_2O_7$ crystal. Supplementary Fig. 1a shows an image of the sample used for these measurements mounted on the CS200T strain cell. The black, bar-shaped sample, seen in the centre of the image, is mounted between titanium sample plates over a distance of around 1 mm. At zero strain and 47.7 K, in the centre of the ICC phase, this sample produces the diffraction pattern shown in Supplementary Fig. 1b. A remnant central peak at $(0, 0, 1)$, which arises from the collinear AFM_a and AFM_b phases [1], can be seen due to slight temperature and strain gradients through the probed region of the sample. Either side of this, satellite peaks are visible at $(\pm\delta, 0, 1)$ with $\delta \approx 0.023$ due to the ICC phase [2].

We tracked the intensity of these peaks under strain. In Fig. 1c of the main text, we present the results obtained at 47.7 K, which show a suppression of the satellite peaks and concomitant increase in intensity of the commensurate peak under both positive and negative strain. In Supplementary Fig. 1c we show the results of similar measurements performed at 46.2 K where we are in the AFM_b phase at zero strain. We see a relative insensitivity

of the peaks to tensile strain, whereas compressive strains beyond $\Delta L/L <$ -0.3% lead to a strong decrease of the $(0, 0, 1)$ intensity and simultaneous increase of the $(\pm\delta, 0, 1)$ intensities. Supplementary Fig. 1d shows results at 48.5 K, where we are in the AFM_a phase at zero strain. Here, we see the opposite behaviour, with little change under compression and an exchange of intensity above 0.2% tensile strain. These data confirm the conclusion in the main text that increasing strain (i.e. from compressive to tensile) drives transitions from AFM_a to ICC, and then from ICC to AFM_b.

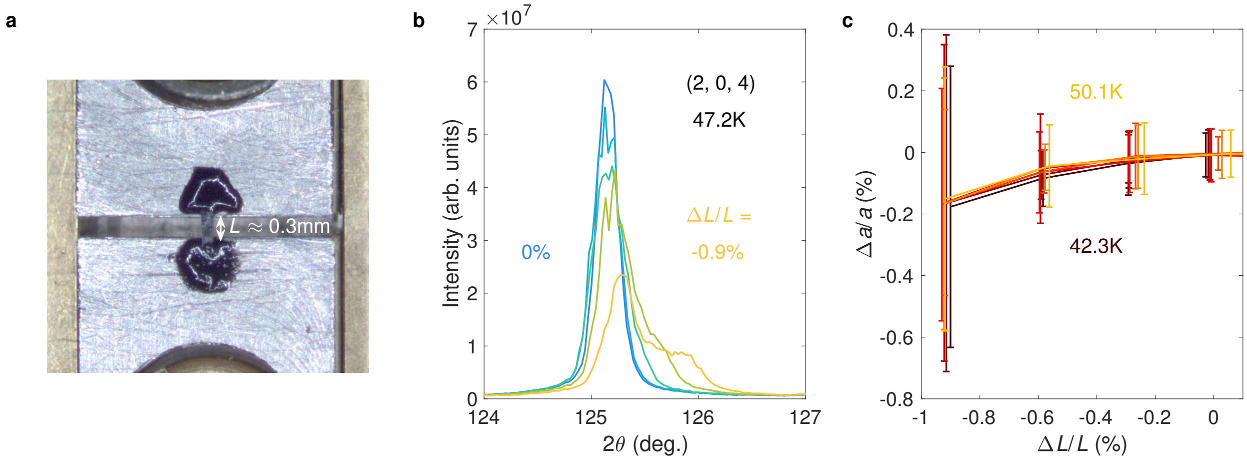

Supplementary Fig. 2. **X-ray scattering with stress along the *a*-axis.** **a** Image of the $Ca_3Ru_2O_7$ sample mounted over a distance $L \approx 0.3$ mm between the sample plates of the CS100 cell. **b** 2θ scans of the structural $(2, 0, 4)$ Bragg peak as a function of applied strain with stress along the a-axis. **c** True strain along the a-axis as a function of applied strain at a range of temperatures through the SRT. Errors are standard deviations.

SUPPLEMENTARY NOTE 2: STRUCTURAL ANALYSIS

Our x-ray scattering setup enabled a detailed study of how the crystal structure of $Ca_3Ru_2O_7$ responds to applied stress. We measured samples with stress applied along both the **a** and **b** directions, with data from the latter shown in Fig. 2 of the main text. Supplementary Fig. 2a shows an image of a sample stressed along **a** mounted on the CS100 cell, from which the phase diagram in Fig. 3a of the main text was constructed. To maximise beam access, the sample is mounted on top of raised sample plates (across a gap of around 0.3 mm), which causes a slight bending of the sample under strain and appears to impair our ability to transmit tensile strain, as discussed below.

2θ scans of the structural $(2, 0, 4)$ Bragg peak of this sample are shown in Supplementary Fig. 2b at various applied strains. We see a shifting of the peak under compressive strain due to the expected change in the lattice parameters. Unlike for the sample stressed along **b** (see Fig. 2b of the main text), however, we also see a significant broadening of the peak under strain, with a shoulder forming on the high-2θ side. This indicates that the magnitude of the strain is inhomogeneous in the probed region. We attribute this to preexisting defects in the sample, as a number of Bragg peaks were asymmetric before any strain was applied.

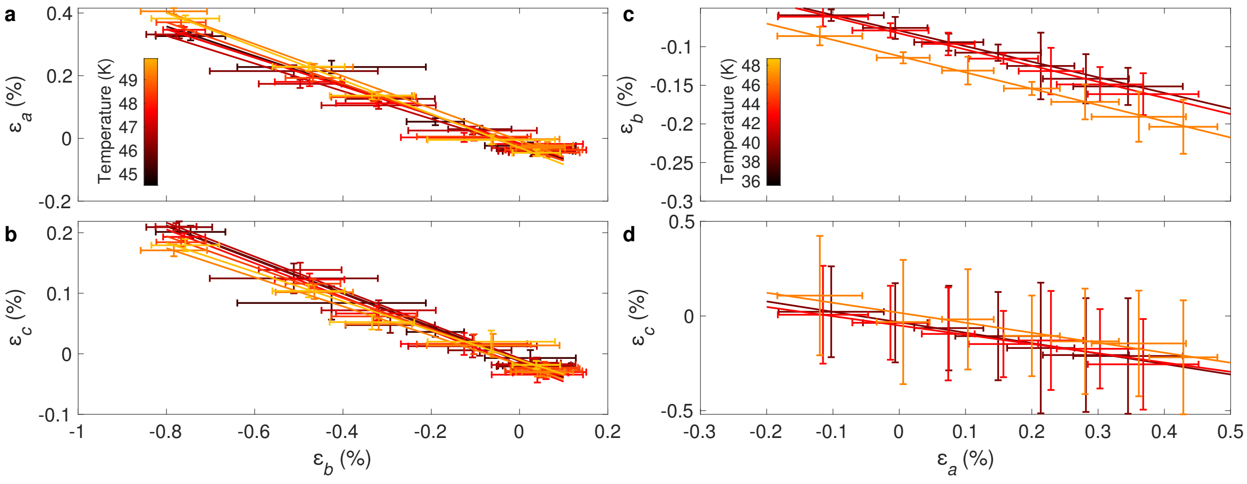

Supplementary Fig. 3. **Poisson ratios.** **a–b** Induced strain along the a- and c-axes as a function of strain along the b-axis at a range of temperatures. **c–d** Induced strain along the b- and c-axes as a function of strain along the a-axis at a range of temperatures. The solid lines are linear fits at each temperature used to determine the Poisson ratios, and all errors are standard deviations.

Given the strain inhomogeneity, we used the centre of mass of the peaks to determine the true strain, shown in Supplementary Fig. 2c.

Measurement of multiple Bragg peaks as a function of strain allows us to determine the strain along all three crystallographic directions. Supplementary Fig. 3 shows the relationship between the orthogonal strains, which are found to be linear at all temperatures (note that the data in Supplementary Fig. 3c–d are obtained from a different sample than that in Supplementary Fig. 2). The Poisson ratios, shown in Fig. 2d of the main text, are determined from the slope of linear fits to these dependences.

As well as probing average changes in the lattice parameters, we can leverage the large area detector at I16 to efficiently map the peaks in full 3D reciprocal space, giving insight into stress-induced variations of the lattice through the probed region. The left panels of Supplementary Fig. 4 show 2θ, h, k and l cuts through three structural Bragg peaks at a range of applied strains (for the sample stressed along **b** at 44.5 K). Alongside the shifting of the peaks, a splitting can be seen in the k–l plane, with a distribution of intensity between the extremal split components. The positions of the main components, converted into reciprocal Ångstroms, are plotted in the right panel for zero and maximum compressive strain. Under compression, the split peaks lie on arcs in reciprocal space and are separated

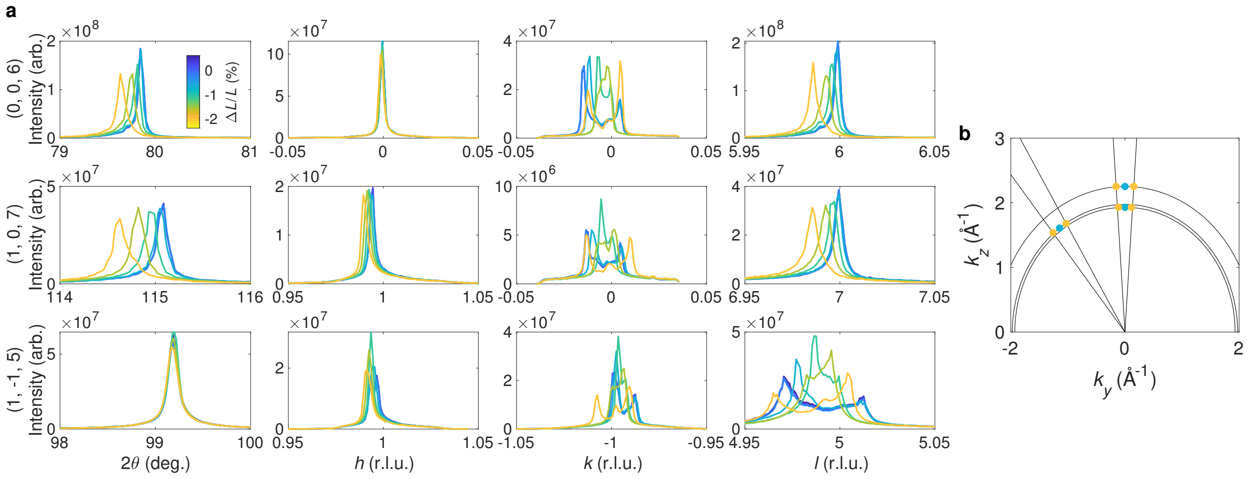

Supplementary Fig. 4. Sample bending. **a** Various reciprocal-space cuts through the structural $(0, 0, 6)$ (top row), $(1, 0, 7)$ (central row), and $(1, -1, 5)$ (lower row) peaks at a range of applied strains with stress along the b-axis at 44.5 K. The left-most column shows 2θ scans, the next column h scans, then k scans, and finally l scans. **b** Projected k–l reciprocal space map in inverse Ångstroms, showing the position of the split peaks at zero (blue) and maximum compressive (yellow) applied strain. The black lines depict the arcs and radial lines on which the split peaks lie.

by approximately the same angle. The splitting can therefore be naturally explained by a bending of the sample under stress, which occurs due to the asymmetric mounting that transmits the strain mostly through the lower face of the sample. Despite the bending, the peaks do not broaden in 2θ beyond our angular resolution, showing that the magnitude of the strain remains fairly homogeneous through the probed region. Interestingly, the peak splitting occurs for both tensile and compressive applied strain. This indicates that our inability to transmit tensile strain to the sample is not due to it fully fracturing (which would result in no response to tensile strain), but must be due to some other mechanism of strain relaxation in the sample and/or epoxy. We saw this same behaviour (peak splitting but no shift in 2θ under tensile strain) in all samples mounted asymmetrically (including in the resistivity measurements described below), but in the neutron measurements with the sample sandwiched symmetrically between sample plates we see no bending and are able to transmit tensile strain.

These results highlight the importance of proper structural analysis when performing strain experiments. The majority of strain studies to date have relied on the *applied* strain

(i.e. the separation of the sample plates) as a proxy for the *true* strain (often with a constant factor introduced to account for deformation of the epoxy on the basis of finite element analysis) [3–7]. Had we done the same, we would have significantly overestimated the compressive strain, been unsure why the magnetic phases did not respond to tensile strain, had no ability to determine the Poisson ratios, and been unaware of the sample bending.

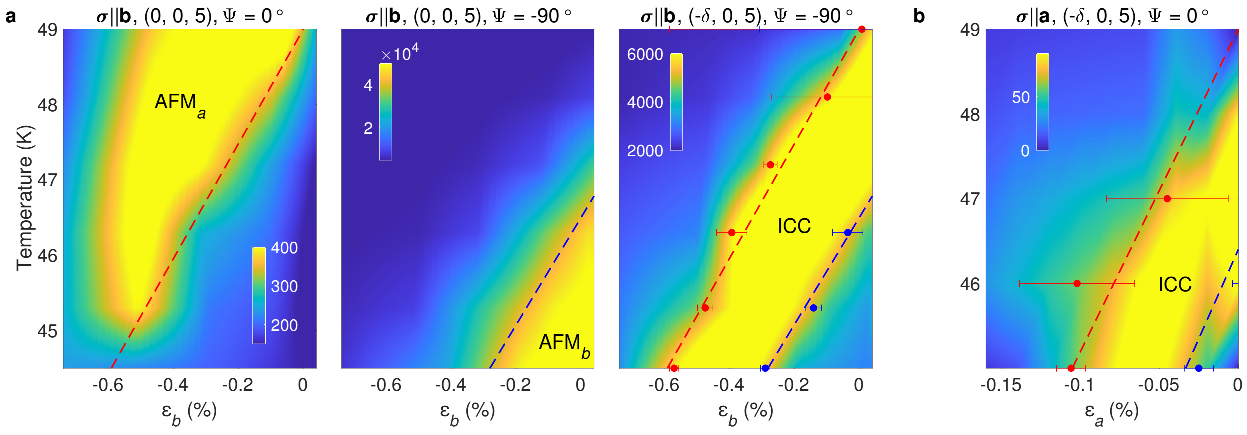

Supplementary Fig. 5. **Phase diagrams.** **a** Phase diagrams for stress applied along the b-axis. In the left panel the colourmap shows the integrated intensity of the commensurate $(0, 0, 5)$ peak at an azimuth of 0° (sensitive to the component of the moment along the a-axis), the central panel shows the intensity of the $(0, 0, 5)$ peak at an azimuth of $-90°$ (sensitive to the moment along the b-axis), and the right panel shows the intensity of the satellite $(-\delta, 0, 5)$ peak. **b** Phase diagram for stress applied along the a-axis, with the colourmap showing the intensity of the satellite $(-\delta, 0, 5)$ peak. The points with error bars (standard deviations) are fits to the ICC phase boundaries defined by the positions of half-maximum intensity, and the dashed red (blue) lines are linear fits through these points.

SUPPLEMENTARY NOTE 3: TEMPERATURE-STRAIN PHASE DIAGRAMS

To construct the magnetic phase diagrams shown in Fig. 3 of the main text, we tracked the intensities of the $(0, 0, 5)$ (at two azimuths) and $(\delta, 0, 5)$ peaks during strain sweeps at multiple temperatures. These intensities are plotted as colourmaps in Supplementary Fig. 5a–b for stress applied along **b** and **a** respectively. From left to right, the three panels in Supplementary Fig. 5a show the intensity of the commensurate $(0, 0, 5)$ peak at an azimuth of $\Psi = 0°$, sensitive to the AFM$_a$ phase, the $(0, 0, 5)$ at an azimuth of $\Psi = -90°$, sensitive to AFM$_b$, and the $(-\delta, 0, 5)$ satellite peak, sensitive to the ICC phase [2]. The AFM$_b$ phase can be seen in the lower right corner of the phase diagrams, disappearing with increasing temperature or compressive strain to be replaced by the ICC and then the AFM$_a$ phases. One unexpected feature in the leftmost panel is the suppression of commensurate intensity above $|\varepsilon_b| \approx 0.6\%$ compressive strain at all temperatures. The reason for this loss of intensity

requires further investigation, but it may be connected to the bending of the sample at high strain, which could disrupt the long-range order of the already-weak AFM_a phase [the (0, 0, 5) intensity is two orders of magnitude lower in the AFM_a phase than the AFM_b]. We note that there is no evidence of a similar suppression in our neutron results, where the bending does not occur.

Supplementary Fig. 5b similarly shows the intensity of the $(-\delta, 0, 5)$ satellite for stress along **a**. As discussed in the previous section, the data quality for this sample was significantly worse than that with stress along **b**, but despite this approximately linear phase boundaries can still be seen. The phase boundaries plotted in Fig. 3 of the main text are determined by finding the strain values at each temperature at which the satellite intensity falls to half its maximum value. These points are shown in red/blue in Supplementary Fig. 5, with error bars purely from fitting of the intensity profile and not accounting for the errors in the strain values. The dashed lines show linear fits through these points.

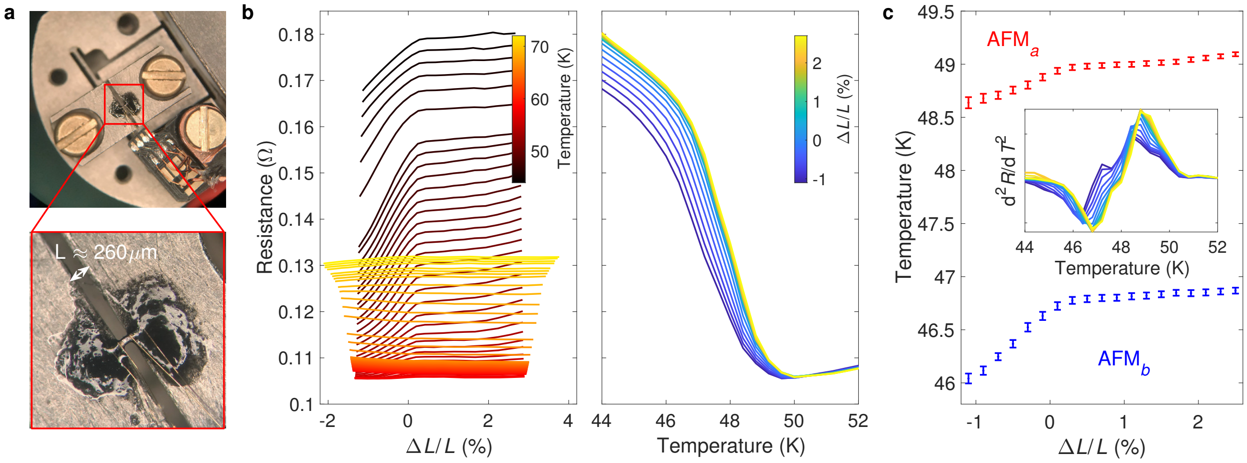

Supplementary Fig. 6. **Transport measurements.** **a** The upper panel shows an image of the CS100 cell set up for resistivity measurements, with the WP100 wiring platform visible on the bottom right. The lower panel shows a close-up of a $Ca_3Ru_2O_7$ sample mounted on the cell, with gold wires contacted to the top surface in a standard four-probe configuration and stress applied along the a-axis. **b** The left panel shows the measured resistance as a function of applied strain at a range of temperatures. The right panel shows the same data plotted as a function of temperature for a range of applied strains. **c** Strain dependence of the transition temperatures, determined from the maxima and minima of d^2R/dT^2 shown in the inset.

SUPPLEMENTARY NOTE 4: TRANSPORT MEASUREMENTS UNDER STRESS

To correlate the changes in the crystal and magnetic structures with the Fermi surface reconstruction, we also performed four-probe resistance measurements under strain in a Quantum Design PPMS. For this we used the same CS100 cell as the x-ray measurements, with the addition of a wiring platform to allow electrical contacts to be made to the sample (see the upper panel in Supplementary Fig. 6a). Four 25 µm-diameter gold wires were contacted along the length of the sample with Dupont 6838 silver paint, and cured at 900 °C for 5 min. This results in mechanically robust contacts that can survive the repeated deformation caused by the applied stress, and it was confirmed that the brief exposure to high temperature does not change the transport properties of the sample. The other ends of the wires were connected with silver epoxy to pads on the wiring platform, which was in turn connected to a standard PPMS puck. The sample was mounted only with sample plates below as in the x-ray measurements, and 10 µm nylon threads were placed in the epoxy below

the sample to prevent electrical contact with the titanium cell (which is grounded during the measurement). The sample was mounted with the current contacts in the unstrained regions covered by epoxy and the voltage contacts in the strained region suspended between the sample plates (see the lower panel in Supplementary Fig. 6a). As the contacts are on the top face of the sample, the current will spread downwards over a length scale $t\sqrt{\rho_c/\rho_{ab}} \sim 100\,\mu\text{m}$ [8]. This is of the same magnitude as the length between the voltage contacts, so there will be a significant contribution to the measured resistivity from ρ_c. Thankfully, ρ_{ab} and ρ_c follow the same trend above 45 K, so this does not affect the determination of the transition temperatures that we are interested in. The CS100 was mounted on a custom PPMS probe supplied by Razorbill Instruments. No temperature sensor was mounted on the cell, but through comparison with the neutron and x-ray data a constant 2 K offset was found and corrected between the cryostat and sample temperatures.

The left panel of Supplementary Fig. 6b shows strain dependences of the in-plane resistance at a range of temperatures for the sample with stress applied along **a**. As we have no access to the true strain in the transport measurements, we plot these dependences as a function of applied strain, $\Delta L/L$. At the lowest temperatures the resistance is highest and we see a clear decrease under compressive applied strain. There is little response to tensile strain, which is expected from the asymmetric mounting as in the x-ray experiments. On warming into the ICC phase, the slope under compression decreases and the resistance appears to increase slightly under high tensile strain $\Delta L/L > 2\%$. This again suggests that the sample is not broken, but that some other mechanism relieves tensile strain. On further warming into the AFM$_a$ phase, the resistance instead begins to increase with compressive applied strain, before again falling under compression above T_N. The same data are plotted in the right panel of Supplementary Fig. 6b as a function of temperature in the vicinity of the ICC phase, showing the resistance curve shifting down in temperature under compression. At zero strain, the boundaries of the ICC phase coincide with changes of slope of the resistance [2]. To check whether this correspondence is maintained under strain, we plot the maxima and minima of d^2R/dT^2 as a function of applied strain in Supplementary Fig. 6c. We again find linear dependences under compression, with gradients that are comparable to those of the magnetic phase boundaries in Supplementary Fig. 5 (after converting true strain to applied strain). This confirms that the electronic and magnetic transitions are

locked together under strain.

---

[1] B. Bohnenbuck, I. Zegkinoglou, J. Strempfer, C. Schüßler-Langeheine, C. S. Nelson, P. Leininger, H.-H. Wu, E. Schierle, J. C. Lang, G. Srajer, S. I. Ikeda, Y. Yoshida, K. Iwata, S. Katano, N. Kikugawa, and B. Keimer, Phys. Rev. B **77**, 224412 (2008).

[2] C. D. Dashwood, L. S. I. Veiga, Q. Faure, J. G. Vale, D. G. Porter, S. P. Collins, P. Manuel, D. D. Khalyavin, F. Orlandi, R. S. Perry, R. D. Johnson, and D. F. McMorrow, Phys. Rev. B **102**, 180410(R) (2020).

[3] C. W. Hicks, D. O. Brodsky, E. A. Yelland, A. S. Gibbs, J. A. N. Bruin, M. E. Barber, S. D. Edkins, K. Nishimura, S. Yonezawa, Y. Maeno, and A. P. Mackenzie, Science **344**, 283 (2014).

[4] A. Steppke, L. Zhao, M. E. Barber, T. Scaffidi, F. Jerzembeck, H. Rosner, A. S. Gibbs, Y. Maeno, S. H. Simon, A. P. Mackenzie, and C. W. Hicks, Science **355**, eaaf9398 (2017).

[5] P. Malinowski, Q. Jiang, J. J. Sanchez, J. Mutch, Z. Liu, P. Went, J. Liu, P. J. Ryan, J.-W. Kim, and J.-H. Chu, Nat. Phys. **16**, 1189 (2020).

[6] H.-H. Kim, E. Lefrançois, K. Kummer, R. Fumagalli, N. B. Brookes, D. Betto, S. Nakata, M. Tortora, J. Porras, T. Loew, M. E. Barber, L. Braicovich, A. P. Mackenzie, C. W. Hicks, B. Keimer, M. Minola, and M. Le Tacon, Phys. Rev. Lett. **126**, 037002 (2021).

[7] T. Worasaran, M. S. Ikeda, J. C. Palmstrom, J. A. W. Straquadine, S. A. Kivelson, and I. R. Fisher, Science **372**, 973 (2021).

[8] J. M. Bartlett, A. Steppke, S. Hosoi, H. Noad, J. Park, C. Timm, T. Shibauchi, A. P. Mackenzie, and C. W. Hicks, Phys. Rev. X **11**, 021038 (2021).
